# Enabled homolog (ENAH) regulated by RNA binding protein splicing factor 3b subunit 4 (SF3B4) exacerbates the proliferation, invasion and migration of hepatocellular carcinoma cells via Notch signaling pathway

**DOI:** 10.1080/21655979.2021.2023983

**Published:** 2022-01-14

**Authors:** Guoming Deng, Yufeng Luo, Yaoming Zhang, Jinfeng Zhang, Zongyun He

**Affiliations:** a The 2nd Department of Hepatobiliary Surgery, Meizhou People’s Hospital, Meizhou, China; b Guangdong Provincial Key Laboratory of Precision Medicine and Clinical Translational Research of Hakka Population, Meizhou, China; c The 3rd Department of Medical Oncology, Meizhou People’s Hospital, Meizhou, China; d The Department of Hepatology, Meizhou People’s Hospital, Meizhou, China

**Keywords:** Hepatocellular carcinoma, ENAH, SF3B4, Notch signaling pathway

## Abstract

Enabled homolog (ENAH) is an actin-binding protein that implicated in multiple malignant tumors. High ENAH expression has been verified to be associated with poor prognosis in hepatocellular carcinoma (HCC). We aimed to reveal the role of ENAH in HCC and the potential mechanism. ENAH expression in HCC tissues and the prognostic correlation were analyzed by GEPIA2 database. RT-qPCR and Western blot were used to test ENAH expression in HCC cells. Following ENAH silencing, cell proliferation was estimated by CCK-8 and colony formation assays. Transwell and wound healing assays were to assess cell invasion and migration. ENCORI database was to analyze the correlation between ENAH and splicing factor 3b subunit 4 (SF3B4) in HCC tissues, which was then verified by RIP and actinomycin D assay. Then, the expression of Notch signaling-related proteins was detected by Western blotting after ENAH knockdown. Afterward, Notch1 was overexpressed to validate whether ENAH impacted the biological events of HCC cells through mediating Notch signaling. Results revealed that ENAH expression was elevated in HCC tissues and cells and associated with poor prognosis. ENAH deficiency mitigated proliferation, invasion and migration of HCC cells. Mechanistically, ENAH was positively correlated with SF3B4 in HCC tissues. SF3B4 could bind to ENAH mRNA and stabilized ENAH. Besides, ENAH activated Notch signaling. Notch1 up-regulation reversed the influence of ENAH knockdown on biological events of HCC cells. Collectively, ENAH regulated by SF3B4 promoted the development of HCC through activating Notch signaling, which identified ENAH as a potent molecular target for HCC therapy and prognosis.

## Introduction

Primary liver tumor, one of the most frequent malignant tumors, is deemed to threaten human health severely due to its high incidence and mortality rate [1]. Hepatocellular carcinoma (HCC), accounts for almost 90% of all primary liver malignancies, ranks among the commonest type of primary liver tumor [2]. It typically arises in chronic viral hepatitis and cirrhosis largely dependent on the interplay among the host, disease and environmental factors [3]. Moreover, alcohol, tobacco, and obesity significantly increase the risk of HCC [4]. The overall survival rate of HCC patients can be predicted and various therapeutic strategies can be selected based on different tumor stages [5]. For patients in early stage, surgery or radio-frequency ablation is a primary treatment option, while individualized treatments such as systemic treatment or combined radiation therapy are confirmed as effective strategies in HCC patients at advanced stages [6]. Moreover, HCC patients are prone to have a poor prognosis in spite of great advances in the clinical diagnosis and management of HCC in the last decades [7]. Under this circumstance, a better and deeper understanding of the molecular pathology of HCC may help to reveal a novel therapeutic prospect for HCC [8].

Enabled homolog (ENAH), also known as human ortholog of mammalian enabled (hMENA), is a member of the enabled/vasodilator stimulated phosphoprotein (Ena/VASP) gene family [9]. It is an actin-binding protein which is abundant in multiple tumor tissues and participates in the process of gastric cancer, esophageal squamous cell carcinoma and breast cancer. There are some examples highlighting the important role of ENAH in cancers. Wang et al. have elucidated that ENAH expression is increased in gastric cancer and functions as a tumor promoter in the tumorigenesis and development of gastric cancer [10]. He et al. have proposed that ENAH targeted by miR-375 plays the promoting role in the progression of esophageal squamous cell carcinoma [11]. Li et al. have clarified that ENAH can be identified as a possible biomarker for breast cancer prognosis [9]. More importantly, emerging evidence has determined that ENAH is associated with HCC diagnosis and prognosis [12,13]. However, to the best of our knowledge, the concrete role of ENAH in cellular events in HCC and the potential regulatory mechanism are scarcely investigated.

Splicing factor 3b subunit 4 (SF3B4), an RNA-binding protein (RBP) in SF3b splicing factor, is implicated in multiple human diseases via mediating pre-mRNA splicing, translation, transcription and cell signaling [14–16]. Importantly, SF3B4 has been unmasked to be concerned with the diagnosis and prognosis of HCC [17,18]. Liu et al. have further proved that SF3B4 drives cell proliferation and metastasis in HCC [19]. Nevertheless, the interplay between SF3B4 and ENAH in HCC is still unclear. Additionally, it is widely considered that dysregulation of Notch signaling pathway is responsible for the onset and development of tumors [20]. The significance of Notch signaling has been greatly highlighted in diverse kinds of malignancies, such as breast cancer [21], colorectal cancer [22], osteosarcoma [23] and so on. Despite the critical role of Notch signaling in HCC evidenced by quantities of studies [24,25], the speculation whether ENAH regulates Notch signaling in HCC to exert its functions remains to be validated.

The present study was to explore the effects of ENAH on proliferation, invasion and migration of HCC cells and identify the latent regulatory mechanism of ENAH related to SF3B4 and Notch signaling in HCC. The findings of the present study may provide new insight into HCC development and identify a valuable molecular target for HCC diagnosis and treatment in the future.

## Materials and methods

### Bioinformatics tools

ENAH expression in HCC tissues and the correlation between ENAH and the overall survival rate of HCC patients were both analyzed by Gene Expression Profiling Interactive Analysis (GEPIA)2 database (http://gepia2.cancer-pku.cn). The interaction between SF3B4 and ENAH in HCC was analyzed by The Encyclopedia of RNA Interactomes (ENCORI) database (https://starbase.sysu.edu.cn/). Linkedomics database (http://www.linkedomics.org) was employed to conduct Gene Set Enrichment Analysis (GSEA) of ENAH in The Cancer Genome Atlas (TCGA)-liver hepatocellular carcinoma (LIHC) dataset.

### Cell culture

Human HCC cell lines (HCCLM3, HuH-7 and SNU-387) and normal human liver epithelial cell line (THLE-3) were selected for this study. HCCLM3, HuH-7 and THLE-3 cells obtained from the Chinese Academy of Sciences Cell Bank (Shanghai, China) were maintained in Dulbecco’s modified Eagle’s medium (DMEM; Invitrogen, Thermo Fisher Scientific, Inc.). SNU-387 cell line purchased from American Type Culture Collection (ATCC; Manassas, VA, USA) was inoculated in Roswell Park Memorial Institute (RPMI)-1640 Medium (HyClone, South Logan, UT, USA). All mediums were supplemented with 10% fetal bovine serum (FBS) and 1% antibiotics and kept under the condition of 5% CO_2_ and 37°C.

### Cell transfection

To knockdown ENAH or SF3B4, the specific short hairpin RNAs (shRNAs) targeting ENAH (shRNA-ENAH-1/2) or SF3B4 (shRNA-SF3B4-1/2) which regarded shRNA-NC as the negative control were designed and synthesized by Genechem (Shanghai, China). In addition, plasmids (GenePharma, Shanghai, China) carrying Notch1 gene (Ov-Notch1) or empty vector (Ov-NC) were transfected for overexpression of Notch1. Above plasmid transfection was conducted following the instructions of Lipofectamine 2000 (Invitrogen, CA, USA). After 48 h of transfection, cells were harvested for subsequent experiments.

### Reverse transcription-quantitative PCR (RT-qPCR)

Total RNAs were isolated from indicated cells using Trizol reagent (Invitrogen, Carlsbad, CA, USA) and then subjected to reverse transcription by M-MLV Reverse Transcriptase (Invitrogen). The obtained complementary DNA (cDNA) then went through PCR with SYBR premix EX TAQ II (Takara, Dalian, China) on the ABI Prism 7700 Sequence Detection system (Thermo Fisher Scientific, Inc.). Gene expression was quantified with the adoption of 2^−ΔΔCt^ methods with GAPDH as the internal reference gene [26].

### Cell counting kit-8 (CCK-8) assay

SNU387 cells (3000 cells/well) were inoculated into 96-well plates and incubated at 37°C for 24, 48, 72 h, respectively. Then, 10 μl CCK-8 solution was added into each well at each time point. After incubation for another 4 h, the absorbance at 450 nm was measured with a microplate reader (ThermoFisher Scientific, Waltham, MA, USA).

### Colony formation assay

Transfected cells (1x10^3^ cells) were seeded in 6-well plates and cultured in complete RPMI-1640 medium for 14 days. After fixed in 4% paraformaldehyde for 15 min at room temperature, cells were stained with 0.1% crystal violet for 5 min at room temperature. The number of colonies was counted manually using a light microscope (Olympus Corporation).

### Wound healing assay

Cells were seeded into 6-well plates at the density of 4 × 10^5^ cells/well. When cells reached 70–80% confluence, a 100-µl pipette tip was used to create an artificial wound in the confluent cell monolayer. The cell migration rate at 0 h and 24 h was observed and calculated using a light microscope (Olympus Corporation).

### Transwell invasion assay

The invasive ability of SNU387 cells was estimated with the adoption of transwell method. Generally, the upper chamber of the transwell (BD) was precoated with Matrigel (BD, Franklin Lakes, NJ, USA). SNU387 cells (2x10^5^/well) were seeded in serum-free medium in the upper chambers. Meanwhile, 500 μL RPMI-1640 Medium containing 10% FBS was added into the lower chambers. The invaded cells were stained with 0.1% crystal violet after incubation for 24 h before being counted by a microscope (Olympus, Tokyo, Japan).

### RNA immunoprecipitation (RIP) assay

RIP assay was carried out with the application of EZ-Magna RIP kit (Millipore, MA, USA). SNU387 cells were lysed in the RIP Lysis Buffer and cell lysates were immunoprecipitated with anti-SF3B4 (Abcam) and anti-IgG (Abcam) antibodies. Finally, RT-qPCR was to analyze the extracted RNA complexes.

### Actinomycin D assay

To test RNA stability, transfected SNU387 cells were collected and administrated with 5 µg/ml actinomycin D (Act D; Sigma-Aldrich) for 4 h, 12 h and 24 h, respectively. RNA was extracted with TRIzol reagent (Invitrogen). Finally, gene expression was measured by RT-qPCR and Western blot analysis.

### Western blot analysis

Total protein extracts were obtained with radioimmunoprecipitation assay (RIPA) lysis buffer (Beyotime) and then quantified by a bicinchoninic acid (BCA) kit (Beyotime). The proteins separated by sodium dodecyl sulfate-polyacrylamide gel electrophoresis (SDS-PAGE) on a 12% gel were transferred onto polyvinylidene fluoride (PVDF) membranes, followed by being impeded in 5% skimmed milk at room temperature for 2 h. Subsequently, the membranes were incubated with primary antibodies against ENAH (Abcam, 1:1000, ab124685), Ki67 (Abcam, 1:5000, ab254123), proliferating cell nuclear antigen (PCNA) (Abcam, 1:1000, ab92552), SF3B4 (Abcam, 1:2000, ab183483), matrix metallopeptidase 2 (MMP2) (Abcam, 1:1000, ab92536), matrix metallopeptidase 9 (MMP9) (Abcam, 1:1000, ab76003), Notch1 (Abcam, 1:1000, ab52627), hes family bHLH transcription factor 1 (HES1) (Abcam, 1:1000, ab108937), mastermind like transcriptional coactivator 1 (MAML1) (Abcam, 1:1000, ab155786) and GAPDH (Abcam, 1:1000, ab8245) overnight at 4°C and further incubated with HRP-conjugated secondary antibody (Abcam, 1:1000; ab7090) at room temperature for 2 h. The resulting bands were visualized under enhanced chemiluminescence (MilliporeSigma). Protein levels were quantified using Image J software (version 1.46; National Institutes of Health).

### Statistical analysis

Experimental data were analyzed by GraphPad Prism 8.0 software (GraphPad Software, San Diego, CA, USA) and expressed as mean ± standard deviation (SD). Student’s t-test was applied to compare differences between two groups. Differences among three and more than three groups were compared by one-way analysis of variance (ANOVA) followed by Tukey’s post hoc test. P < 0.05 was deemed as statistically significant.

## Results

### ENAH expression is elevated in HCC tissues and cells

**Figure 1.**
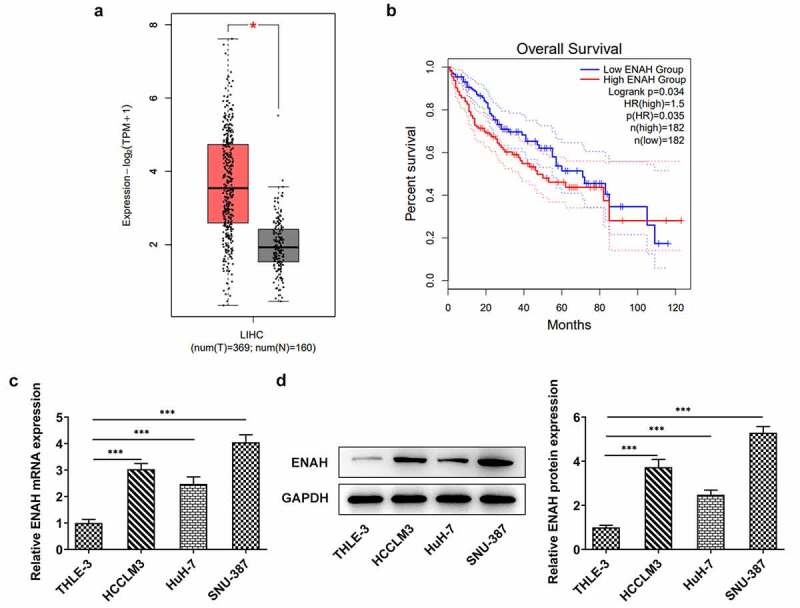
ENAH expression is elevated in HCC tissues and cells. GEPIA2 database detected ENAH expression (a) in HCC tumor tissues and (b) predicted the correlation between ENAH and the overall survival rate of HCC patients. (c) RT-qPCR and (d) Western blot analysis tested ENAH expression in HCC cells. ***P < 0.001. ENAH, Enabled homolog.

Compelling evidence indicates that ENAH is associated with HCC diagnosis and prognosis [12,13]. To measure the concrete role of ENAH in HCC, ENAH expression in HCC tissues was firstly examined by GEPIA2 database. As displayed in Figure 1a, by Student's t-test analysis, ENAH was discovered to be distinctly up-regulated in HCC tumor tissues in comparison with normal tissues. Moreover, the results from survival analysis also suggested that high expression of ENAH predicted a poorer prognosis of HCC patients (Figure 1b). In addition, through RT-qPCR and Western blot analysis, the up-regulation of ENAH in HCC cell lines (HCCLM3, HuH-7 and SNU-387) was noticed compared with that in normal liver epithelial cell line (THLE-3) (Figure 1c-d). Further, ENAH exhibited the highest expression in SNU-387, therefore SNU-387 cell line was chosen for the subsequent experiments. All above results implied that the aberrant expression of ENAH might be involved in the process of HCC.

### Inhibition of ENAH abrogates the proliferation of SNU-387 cells

**Figure 2.**
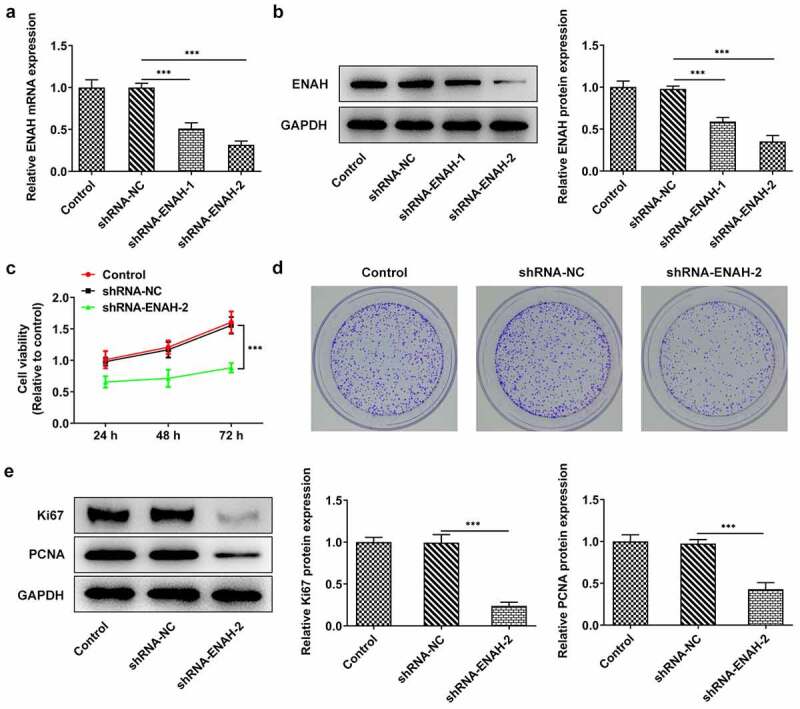
Inhibition of ENAH abrogates the proliferation of SNU-387 cells. (a) RT-qPCR and (b) Western blot analysis tested ENAH expression in HCC cells after transfected with shRNA-ENAH-1/2. (c) CCK-8 and (d) colony formation assay measured HCC cell proliferation. (e) Western blot analyzed the protein levels of Ki67 and PCNA. ***P < 0.001. ENAH, Enabled homolog. PCNA, proliferating cell nuclear antigen.

For the purpose of evaluating the concrete function of ENAH on the malignant phenotypes of HCC cells, ENAH was knocked down in SNU-387 cells by transfection of shRNA-ENAH-1 and shRNA-ENAH-2 and the interference efficiency was tested by RT-qPCR and Western blot analysis (Figure 2a-b). It was observed that ENAH expression was greatly lessened after transfection of shRNA-ENAH-1 and shRNA-ENAH-2 and shRNA-ENAH-2 was selected for the following loss-of-function assays for it showed the best knockdown efficiency. Then, CCK-8 assay demonstrated that after ENAH was silenced, the viability of SNU-387 cells was obviously suppressed (Figure 2c). Colony formation assay also revealed that transfection of shRNA-ENAH-2 also impeded the proliferation of SNU-387 cells (Figure 2d). In addition, the protein levels of proliferation markers including Ki67 and PCNA were analyzed by Western blot and the results manifested that when ENAH was down-regulated, Ki67 and PCNA protein levels were both cut down (Figure 2e). In a word, ENAH deficiency played the suppressive role in HCC cell proliferation.

### ENAH deficiency alleviates cell invasion and migration in HCC

**Figure 3.**
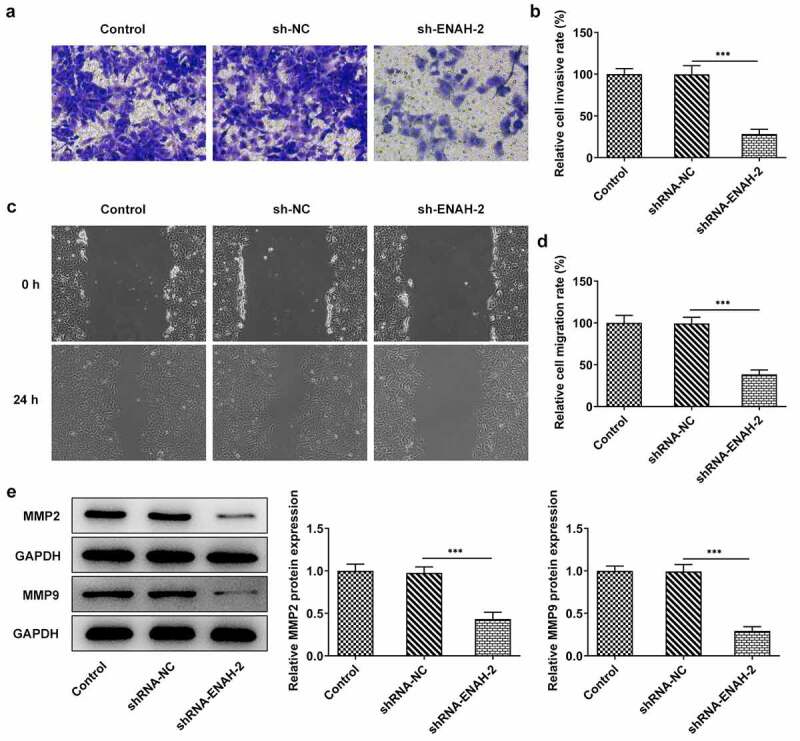
ENAH deficiency alleviates cell invasion and migration in HCC cells. (a-b) Transwell assay was to evaluate the invasive ability of SNU-387 cells. (c-d) Wound healing assay was to estimate cell migration in HCC. (e) MMP2 and MMP9 expression were examined by Western blot analysis. ***P < 0.001. ENAH, Enabled homolog. MMP2, matrix metallopeptidase 2. MMP9, matrix metallopeptidase 9.

Aberrant invasion and migration is implicated in the initiation and development of HCC [27,28]. Therefore, the abilities of HCC cell invasion and migration were determined by transwell and wound healing assays, respectively. From transwell assay, it was noticed that the invasive capacity of SNU-387 cells was dramatically attenuated after transfection of shRNA-ENAH-2 (Figure 3(a-b)). Similarly, wound healing assay was to assess cell migration and the experimental results uncovered that ENAH insufficiency hindered the migration of SNU-387 cells (Figure 3(c-d)). Additionally, Western blot analysis disclosed that ENAH down-regulation greatly decreased the expression of MMP2 and MMP9 (Figure 3e). To be summarized, ENAH functioned as a promoter in HCC cell invasion and migration.

### SF3B4 binds to ENAH and stabilizes ENAH mRNA

**Figure 4.**
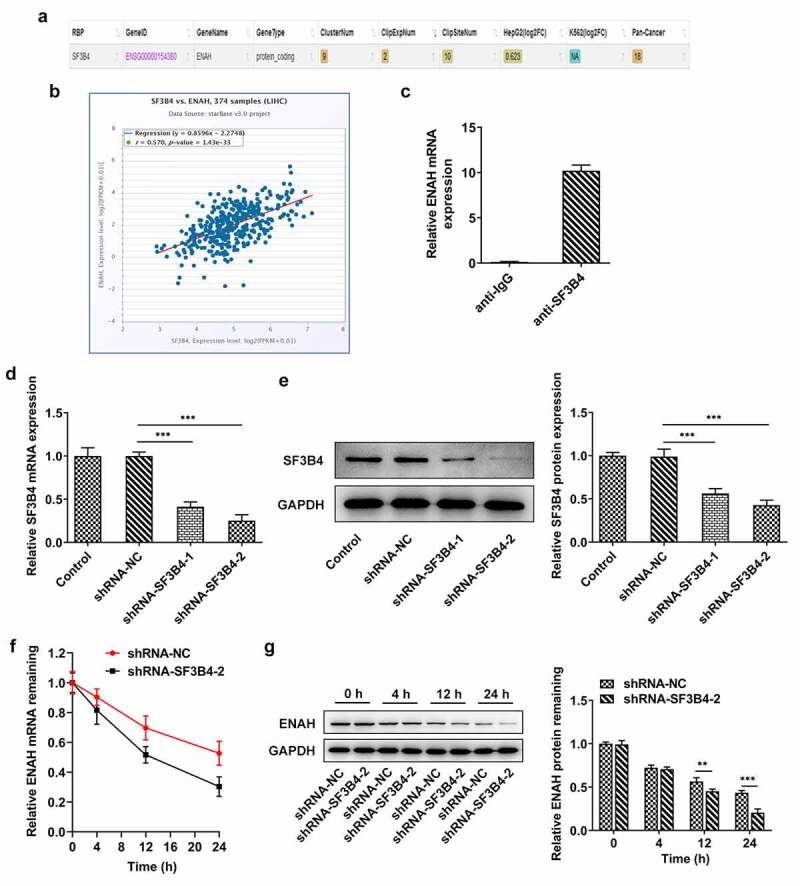
SF3B4 binds to ENAH and stabilizes ENAH mRNA. (a-b) The interaction between ENAH and SF3B4 in HCC was explored by ENCORI database. (c) The enrichment of ENAH in SF3B4 antibody was detected by RIP assay. (d) RT-qPCR and (e) Western blot analysis tested SF3B4 expression in HCC cells after transfected with shRNA-SF3B4-1/2. (f) RT-qPCR and (g) Western blot analysis examined the stability of ENAH mRNA after transfection of shRNA-SF3B4-2. **P < 0.01, ***P < 0.001. ENAH, Enabled homolog. SF3B4, splicing factor 3b subunit 4.

To explore the potential mechanisms of ENAH on the regulation of HCC progression, ENCORI database was used to predict the ENAH associated genes. Based on the results from ENCORI database, ENAH was found to have a positive correlation with SF3B4 in HCC tissues (Figure 4(a-b)). Moreover, RIP assay validated that ENAH mRNA was abundant in anti-SF3B4 group in comparison with that in anti-IgG group (Figure 4c). Subsequently, shRNA-SF3B4-1/2 were transfected into SNU-387 cells to lessen SF3B4 expression and the knockdown efficiency was measured by RT-qPCR and Western blot (Figure 4(d-e)), as a result of which, shRNA-SF3B4-2 was selected for the subsequent experiments due to its prominent interference efficiency. What is more, SNU-387 cells transfected with shRNA-SF3B4-2 plasmids were administrated with 5 µg/ml Act D and the stability of ENAH mRNA was tested at 4 h, 12 h and 24 h, respectively, through RT-qPCR and Western blot. It turned out that inhibition of SF3B4 reduced the stability of ENAH mRNA, especially at 12 h and 24 h (figure 4(f-g)). In brief, SF3B4 could bind to ENAH mRNA and maintained the stability of ENAH.

### ENAH modulates the activation of Notch signaling pathway

**Figure 5.**
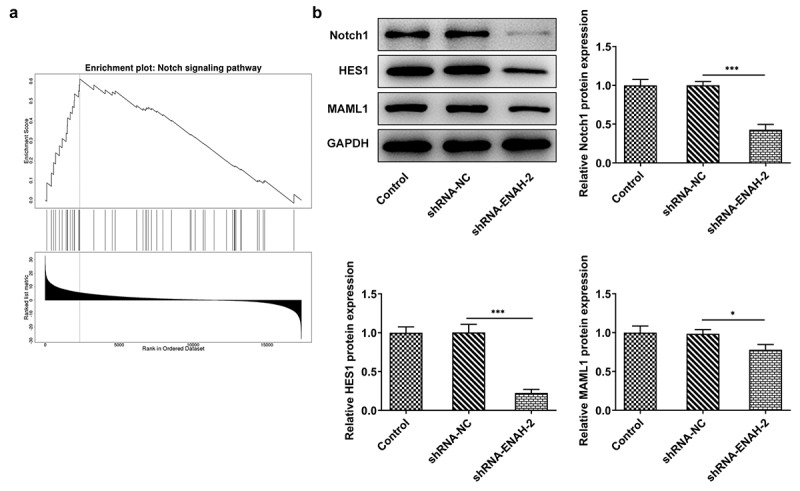
ENAH modulates the activation of Notch signaling pathway. (a) The correlation between ENAH and Notch signaling pathway was detected based on linkedomics database. (b) Western blot analyzed the protein levels of Notch1, HES1 and MAML1. *P < 0.05, ***P < 0.001. Notch1, Notch receptor 1. HES1, hes family bHLH transcription factor 1. MAML1, mastermind like transcriptional coactivator 1.

More intriguingly, GSEA analysis confirmed that ENAH was enriched in Notch signaling pathway and positively regulated the activity of Notch signaling pathway (Figure 5(a)). Also, Western blot analyzed that the protein levels of Notch signaling-related factors including Notch1, HES1 and MAML1 were all decreased after ENAH was silenced (Figure 5(b)). To be concluded, ENAH was an active regulator of Notch signaling pathway.

### Notch1 up-regulation reverses the suppressive role of ENAH silencing in HCC cell proliferation

**Figure 6.**
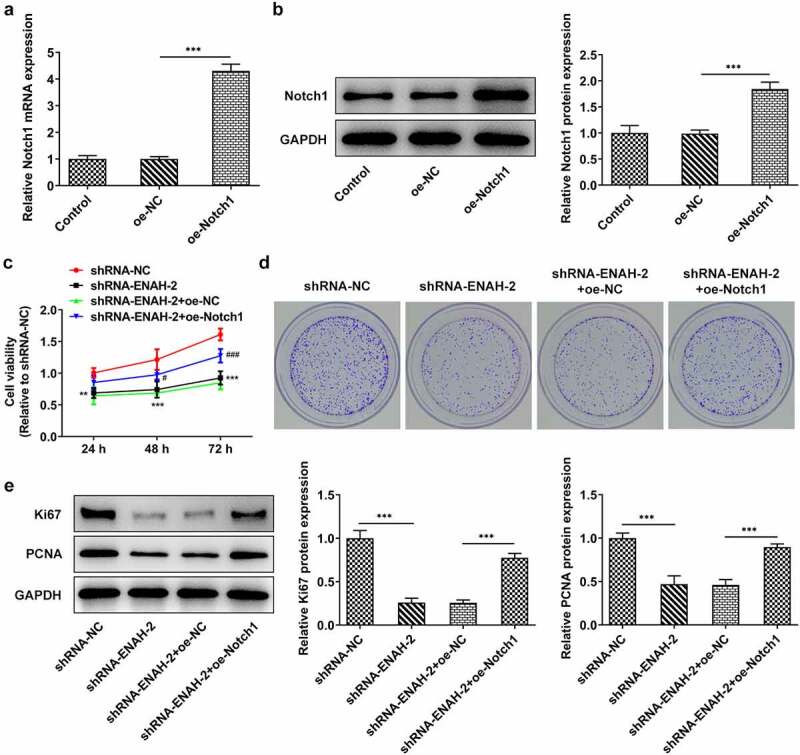
Notch1 up-regulation reverses the suppressive role of ENAH silencing in HCC cell proliferation. (a) RT-qPCR and (b) Western blot analyzed Notch1 expression after transfection of oe-Notch1 plasmids. ***P < 0.001. (c) CCK-8 and (d) colony formation assay measured HCC cell proliferation. ***P < 0.001 vs. shRNA-NC; ^###^P < 0.001 vs. shRNA-ENAH-2+ oe-NC. (e) Western blot analyzed the protein levels of Ki67 and PCNA. ***P < 0.001. ENAH, Enabled homolog. PCNA, proliferating cell nuclear antigen. Notch1, Notch receptor 1.

To verify that ENAH participated in the progression of HCC via regulating Notch signaling pathway, rescue assays were performed. First of all, Notch1 expression was elevated by transfection of oe-Notch1 plasmid and the overexpression efficiency was tested by RT-qPCR and Western blot analysis (Figure 6(a-b)). CCK-8 and colony formation assays elucidated that overexpression of Notch1 partially rescued the suppressive effect of ENAH down-regulation on HCC cell proliferation (Figure 6(c-d)). Western blot further analyzed that ENAH down-regulation reduced the protein levels of Ki67 and PCNA, while this effect could be restored by up-regulation of Notch1 (Figure 6e). Collectively, Notch1 could restore the suppressed SNU-387 cell proliferation caused by ENAH deficiency.

### Notch1 overexpression restores the inhibitory effect of ENAH knockdown on HCC cell invasion and migration

**Figure 7.**
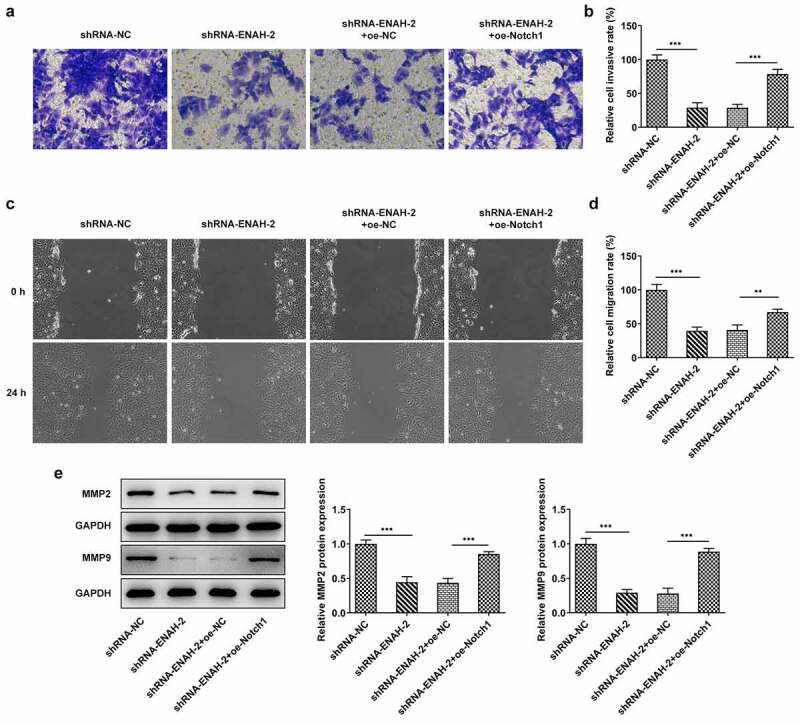
Notch1 overexpression restores the inhibitory effect of ENAH knockdown on HCC cell migration and invasion. (a-b) Transwell assay was to evaluate the invasive ability of SNU-387 cells. (c-d) Wound healing assay was to estimate cell migration in HCC. (e) MMP2 and MMP9 expression were examined by Western blot. **P < 0.01, ***P < 0.001. ENAH, Enabled homolog. MMP2, matrix metallopeptidase 2. MMP9, matrix metallopeptidase 9. Notch1, Notch receptor 1.

To determine whether ENAH affected the invasion and migration of HCC cells through Notch signaling pathway, transwell and wound healing assays were performed again. Transwell assay manifested that the inhibited cell invasion imposed by ENAH knockdown was offset by overexpression of Notch1 (Figure 7(a-b)). The results from wound healing assay also testified that ENAH aggravated HCC cell invasion via regulating Notch1 expression, as evidenced by the finding that Notch1 up-regulation countervailed the abrogated cell migration on account of ENAH inhibition (Figure 7(c-d)). Eventually, the decreased MMP2 and MMP9 expression caused by ENAH interference were both restored by Notch1 elevation (Figure 7e). Taken together, ENAH abrogation ameliorated HCC cell migration and invasion via down-regulating Notch1 expression.

## Discussion

It is well documented that HCC is a heterogeneous disease with high tumor recurrence or metastasis rate [29]. More importantly, changes in the structure or expression of tumor suppressor genes or oncogenes in HCC make the clinical and molecular pathogenesis of HCC more complex [30]. Aberrant malignant cellular events including proliferation, migration and invasion may be implicated in the initiation and development of HCC [27]. In detail, Ki67 and PCNA are two nuclear proliferation markers in tumors [31]. MMP2 and MMP9 are confirmed as crucial regulators of tumor metastasis [32,33]. As reported, ENAH is identified as a tumor promoter in gastric cancer [34], esophageal carcinoma [35] and breast cancer [36]. Moreover, in the literature, ENAH can be used as an independent prognostic marker for HCC [12,13]. Consistent with these findings, ENAH was found to be dramatically up-regulated in HCC tissues and high expression of ENAH predicted a poorer prognosis of HCC patients. Through functional experiments, it was observed that ENAH depletion impeded cell proliferation and decreased the protein levels of Ki67 and PCNA. In addition, silencing of ENAH weakened the migratory and invasive abilities of HCC cells and significantly attenuated MMP2 and MMP9 expression.

Splicing factor SF3b is a multiprotein complex which is engaged in the assembly and activation of spliceosome [37]. Recent researches have exposed that SF3b and its components are essential for diverse kinds of molecular and cellular events [14]. SF3B4, an RNA-binding protein in SF3b, exerts vital functions in the translation of secretory proteins [15]. Emerging evidence has underlined the pivotal regulatory roles of SF3B4 in tumors. SF3B4 has been testified as an oncogene in esophageal squamous cell carcinoma [38]. What is more, SF3B4 is determined as a potential diagnostic marker for HCC and contributes to the progression of HCC [17–19]. On the contrary, Zhou et al. have revealed that SF3B4 is down-regulated and plays the suppressive role in the development of pancreatic cancer [39]. Our present study validated that SF3B4 had a positive correlation with ENAH in HCC. Also, SF3B4 had a strong affinity with ENAH. Besides, interference of SFEB4 led to the decrease on the stability of ENAH mRNA.

Growing evidence has illustrated that dysregulation of signaling pathways is greatly implicated in the tumorigenesis and development of HCC [40,41]. Notch signaling pathway is known as a highly conserved signaling pathway and participates in cancer metastasis [42]. At the same time, numerous studies have figured out that Notch signaling pathway mediates malignant cell phenotypes to drive the progression of HCC [25]. AML1 is the main coactivator of Notch signaling pathway and has a distinct role in the modulation of Notch signaling [43]. Similarly, transcription factor HES1, a basic helix-loop-helix transcriptional repressor, is an effector of Notch signaling pathway [44]. As a member of Notch family which is comprised of highly conserved transmembrane receptors, Notch1 is an important determinant in multiple physiologic processes of normal cells [45]. Accumulating reports have emphasized the regulatory roles of Notch1 in malignant cancers, such as tongue cancer [46], nasopharyngeal carcinoma [47], bladder cancer and so on [48]. More interestingly, the role of Notch1 in HCC has gone through extensive investigation. For instance, Chen et al. have proposed that Notch1 is identified as a promoter in HCC through aggravating vasculogenic mimicry [49]. Han et al. have uncovered that Notch1 targeted by miR-449a exacerbates cell invasion and tumor metastasis in HCC [50]. Tian et al. have suggested that Notch1 exerts a great influence on drug resistance in HCC cells [51]. Through GSEA analysis, it turned out that ENAH was greatly accumulated in Notch signaling pathway, which is also evidenced by the decreased protein levels of Notch1, HES1 and MAML1 after ENAH was knocked down. Furthermore, rescue assays validated that insufficiency of ENAH alleviated the proliferation, invasion and migration of HCC cells, whereas this effect was partially counteracted by up-regulation of Notch1.

## Conclusion

To be concluded, ENAH functionally contributed to cell proliferation, invasion and migration of HCC. Mechanistically, SF3B4 as a RBP of ENAH and positively modulated ENAH in HCC. Moreover, ENAH regulated by SF3B4 promoted the development of HCC through activating Notch signaling. All these findings implied that ENAH might be used as a valuable molecular target for HCC diagnosis and prognosis in the future.

## Data Availability

All data generated or analyzed during this study are included in this published article.
